# Two-photon imaging of the trabecular meshwork

**Published:** 2010-05-29

**Authors:** David A. Ammar, Tim C. Lei, Emily A. Gibson, Malik Y. Kahook

**Affiliations:** 1 Department of Ophthalmology, University of Colorado Denver, Aurora, CO; 2 Department of Electrical Engineering, University of Colorado Denver, Aurora, CO; 3 Department of Physics, University of Colorado Denver, Aurora, CO

## Abstract

**Purpose:**

To image the trabecular meshwork (TM) in its native unfixed state using a non-invasive, non-destructive technique.

**Methods:**

Two-photon microscopy (2PM), including two-photon excitation fluorescence (2PEF) and second harmonic generation (SHG), was used to image flat-mounted trabecular meshwork samples from human cadaver eyes. Multiple images were analyzed along the tissue axis (z-axis) to generate a three-dimensional (3D) model of the region.

**Results:**

A lattice of large collagen fibers (~10 µm in diameter) were detected by inherent fluorescence (2PEF) and SHG. There are regions of both tightly overlapping bundles as well as fluid-filled regions visible from the surface of the TM. 3D analysis of multiple images reveals that the open regions deep in the TM penetrate the juxtacanalicular TM (JTM) and connect to the inner wall of Schlemm’s canal (IWSC). These open regions may represent low-resistance fluid pathways between the anterior chamber and Schlemm’s canal (SC).

**Conclusions:**

2PM imaging of the outflow system of the human eye documented collagenous structures solely from inherent optical properties, without addition of an exogenous fluorescent label. 2PM successfully imaged into the TM without the need for fixation, embedding, or histological processing. Deep penetration using advanced optical techniques revealed regions likely representing pores in the IWSC that have been documented by multiple electron microscope studies. Our work reveals that 2PM imaging has potential as a new metric for evaluating the aqueous outflow region of the human eye and is worthy of further exploration.

## Introduction

In the conventional outflow system of the eye, aqueous humor exits the anterior chamber through the trabecular meshwork (TM), passing through Schlemm’s canal (SC) and collector channels (CC) before finally draining into aqueous veins and episcleral vessels. The TM cells, and more specifically the juxtacanalicular TM (JTM) cells, do not function properly in patients with glaucoma [1]. As a focal point for the pathogenesis of glaucoma, measuring differences in structure and function between the normal and diseased TM cells would be of great importance. However, no current clinical instrumentation exists that can image the structure and/or function of TM cells with fine enough resolution to validate this approach as a potential metric for diagnosing or following the progression of glaucoma. Currently, clinical imaging of the TM region of the eye is limited to use of a mirror to visualize the region (a technique known as gonioscopy). This technique allows for the gross detection of surface abnormalities and is not generally relevant for the diagnosis of open-angle glaucoma, which is the main type of glaucoma affecting patients in the United States. Another device used to examine the outflow system of the eyes is optical coherent tomography (OCT). OCT uses computer interpretation of the interference pattern from long-wavelength light to generate cross-sectional images of the eye with a 18 µm axial and 60 µm transverse resolution [2]. A third device used to assess the drainage system of the eye is ultrasound biomicroscopy, which has a similar resolution (~15 µm) [3] as OCT with better ability to detect small density differences. Neither method has the resolution to accurately image the conventional outflow pathway (SC and CC), or the ability to distinguish individual TM cells.

The morphology of the TM has been examined by electron microscopy (EM), with many studies revealing differences in ultrastructure of the TM between normal and glaucomatous eyes [4-10]. Exact agreement of the differences, however, is lacking. In demonstrating the exceptional images created by quick-freeze deep etching, Gong et al. [10] hypothesized that these differences may result from the loss of extracellular matrix in the JTM during the processing steps necessary for conventional scanning EM. We propose that two-photon microscopy (2PM) could be a better method for imaging the TM in its native state, obviating the need for fixation and histological processing.

Traditional one-photon microscopy (1PM), either epifluorescence or confocal microscopy, is based on linear absorption and emission processes where a single photon excites and then is emitted by a fluorophore. In contrast, 2PM is based on nonlinear optical processes that involve more than one photon interacting simultaneously with a target molecule. Since the probability of simultaneous absorption is extremely low, the process only occurs with high photon flux. This is achieved using a high-intensity near infrared laser (Titanium: Sapphire laser) with an extremely short pulse duration (femtosecond) as the excitation source. As a result, 2PM offers intrinsic axial cross-sectioning because the process only occurs at the focus of the objective (where the laser intensity is greatest.) As a result, 2PM offers equivalent resolution as confocal microscopy but does not require the use of a pinhole. An additional advantage of using a near infrared laser source is deeper tissue penetration due to reduced light scattering of the longer wavelengths of light.

2PM includes both two-photon excitation fluorescence (2PEF) and second harmonic generation (SHG). 2PEF is very similar to traditional fluorescence, except two photons of a lower energy are simultaneously absorbed to excite a fluorophore. The excited fluorophore subsequently fluoresces a single photon of the appropriate emission wavelength. 2PEF occurs with both endogenous and exogenous fluorophores. When 2PEF is used to excite endogenous fluorophores such as collagen and elastin it is called two-photon excitation autofluorescence or autofluorescence (AF) in short. Another nonlinear process that occurs with 2PM is second harmonic generation (SHG). SHG can only occur with non-centrosymmetric (asymmetric) macromolecular structures. Macromolecules such as collagen (but not elastin) can simultaneously “scatter” two lower-energy photons as a single photon of twice the energy. SHG signal, therefore, occurs at a distinct wavelength (half the excitation wavelength) and can be separated from tissue autofluorescence using a spectral detector.

In this study we evaluated the ability of 2PM for imaging the TM region of human cadaver eyes. The results of this study show an unevenly distributed collagen network over which TM cells are localized as well as optically clear areas extending to the inner wall of SC (IWSC), likely representing areas of fluid flow exiting the anterior chamber.

## Methods

### Human eyes

Human cadaver eyes were obtained from the San Diego Eye Bank (San Diego, CA). Approval was obtained from the Colorado Multiple Institutional Review Board for the use of human material and the tenets of the Declaration of Helsinki were followed. Informed consent was obtained from donors or relatives for use in research. Eyes were from pseudophakic donors with no history of glaucoma. Ages of donors were 73 and 88 years old.

### Two-photon microscopy imaging

2PM imaging was performed using a confocal microscope (LSM 510 META on Axiovert 200M platform; Carl Zeiss MicroImaging Inc., Göttingen, Germany) with Zeiss 510 control software (AxioVision, upgraded to Zen) equipped with a tunable mode-locked Ti:Sapphire laser (Chameleon Ultra II; Coherent Inc., Santa Clara, CA) operating at 800 nm center wavelength, with 100–200 fs pulses at 80 MHz repetition rate. The excitation source (Ti:Sapphire laser) was focused on the human tissue samples by a LCI “Plan-NeoFluar” 25/0.8 NA objective with a 0.21 mm working distance (Carl Zeiss MicroImaging Inc.). The emitted signal was first passed through a BG39 filter to remove residual excitation laser light. The two signals were separated in the Zeiss META spectral detector with user-defined filter ranges of 388 nm to 409 nm for the SHG signal and 452 nm to 644 nm for autofluorescence. Where applicable, filter ranges were set to 388 nm to 409 nm for the SHG signal, 473 nm to 505 nm for the Hoechst 33342 signal, and 537 nm to 623 nm for autofluorescence. Image stacks were collected and processed in the Zeiss 510 control software. Laser power used was between 16 and 28 percent of 3.5 mW (100%).

### Image analysis

Single plane projections of multiple z-sections shown in the Figures, as well as three-dimensional (3D) reconstructions shown in the supplemental videos were generated using AxioVision/Zen software (Carl Zeiss MicroImaging, Inc.). This software was also used to adjust overall brightness and contrast of the images.

## Results

**Figure 1:**
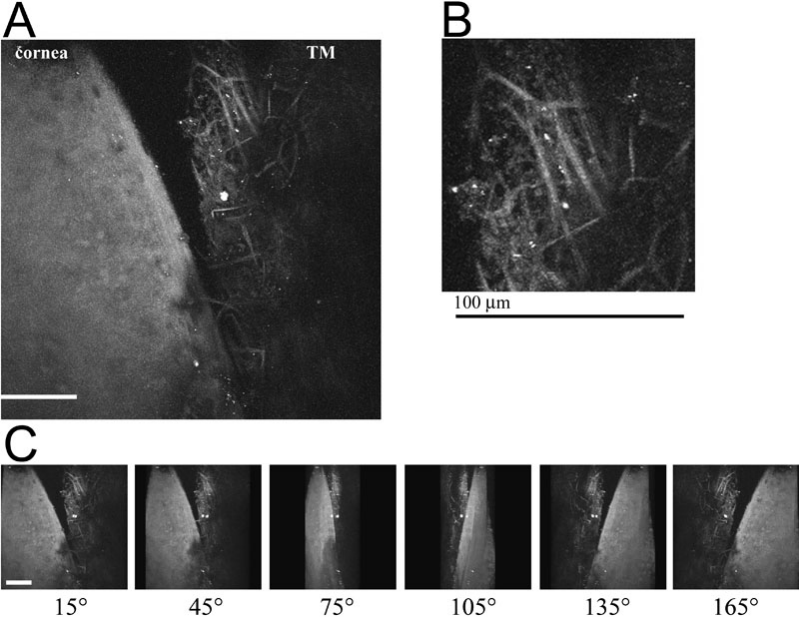
Autofluorescence (AF) of the cornea/TM region of a human eye from a 73-year-old donor. A section of a tissue was flat-mounted with the anterior chamber facing the microscope objective. An AF window (452 nm to 644 nm) was collected from an 800 nm excitation. 100 z-sections were imaged at 1 micron intervals using a 25× objective. **A**: A flattened projection of all z-sections at of the junction of cornea/TM. The curvature of the tissue where the cornea and TM meet (top of **A**) places it beyond the working distance of the objective lens, and therefore it appears as a dark region. **B**: A higher resolution image of the TM region. **C**: Image snapshots of a 3D reconstruction of the cornea/TM region, rotated around the y-axis, shown at intervals of 30°. White/black scale bars=100 µm.

**Figure 2:**
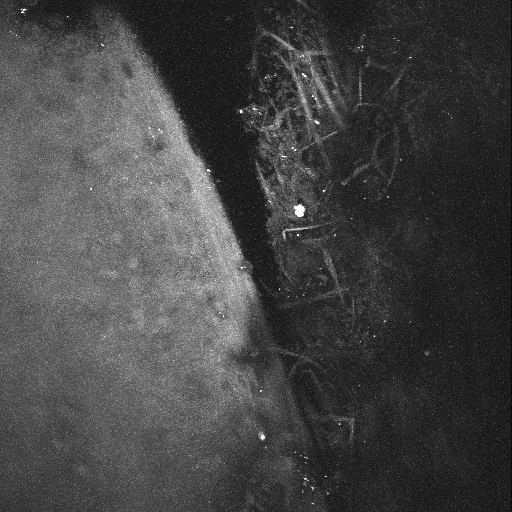
A 3D reconstruction of the cornea/TM region shown in Figure 1. The region of TM/cornea shown in Figure 1 was imaged by AF at 1 µm intervals. A total of 100 z-sections were projected into a 3D-animation with rotation about the y-axis. Rotation begins with a side-on view of the tissue. The AF signal from the collagen ‘beams’ within the TM are quite evident due to the low AF signal from the surrounding fluid spaces. In contrast, the AF signal from the cornea is fairly homogenous.

2PM was performed on a human eye with the TM flat-mounted toward the 25× (0.8 NA) objective lens. With this numerical aperture and an excitation wavelength of 800 nm, the estimated lateral resolution is ~0.6 µm and the longitudinal resolution is ~3 µm. Figure 1 shows images from the aqueous humor face of the TM/cornea. Autofluorescence (AF) from the extracellular matrix was imaged in the TM and cornea in a series of 100 parallel images spaced at 1 µm, and the resulting stack flattened into a single image, shown in Figure 1A. Higher resolution imaging of the collagen within the TM by AF yields the image in Figure 1B. In these flattened projections, the collagen network appears as a meshwork of ~10 µm thick fibers easily visible by their inherent fluorescent properties. Projection of the sections in Figure 1A into 3-dimensions (3D) yields an animation shown in Figure 2; snapshots of this movie were taken every 30° and shown in Figure 1C.

**Figure 3:**
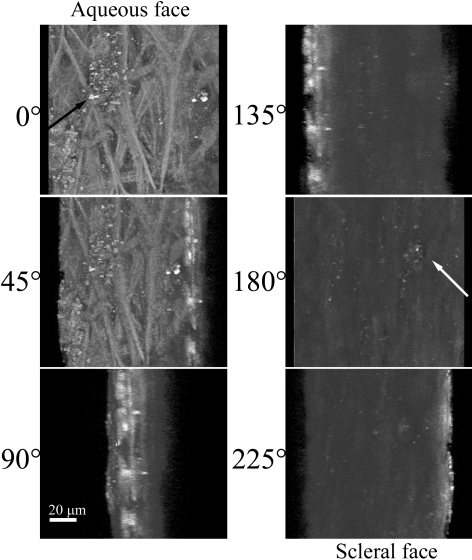
AF of the TM region of a human eye from a 73-year-old donor. Another section TM tissue was flat-mounted with the anterior chamber facing the microscope objective. AF was collected as in Figure 1. Single image snapshots from the 3D reconstruction of the TM region are shown along with corresponding degree of rotation (around the y-axis). 0° represents the aqueous humor face of the TM, while 180° represents the scleral-directed face. A non-fibrous structure present at the aqueous surface (black arrow) can be viewed from the scleral face through an open pore-like structure (white arrow). White scale bar=20 µm.

**Figure 4:**
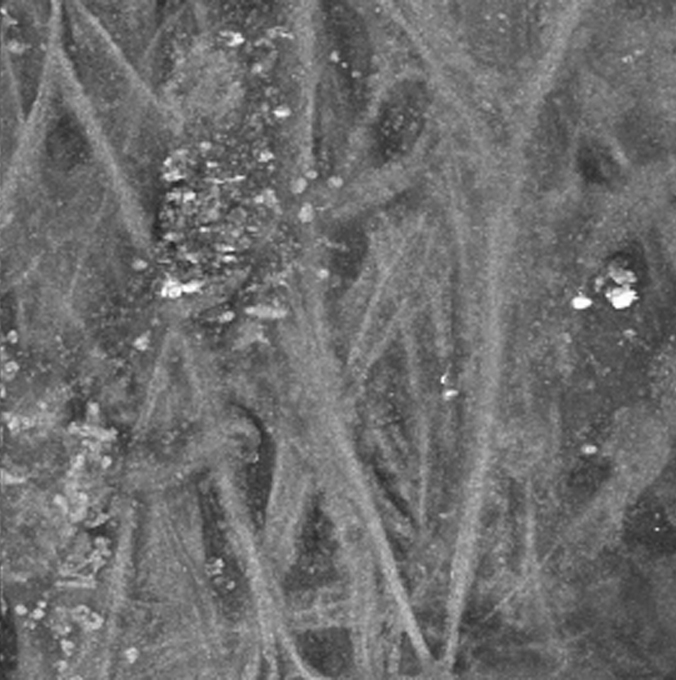
A 3D reconstruction of the TM region shown in Figure 3. The region of TM shown in Figure 3 was imaged by AF at 1 µm intervals. A total of 75 z-sections were projected into a 3D-animation with rotation about the y-axis. The AF signal from the collagen within the TM reveals a meshwork of collagen structures interwoven with what appears to be fluid spaces. The animation begins with a view of the aqueous face of the TM; rotation by 180 degrees reveals an open pore-like structure that penetrates the entire thickness scanned. The non-fibrous structure present at the aqueous surface can be viewed through the pore.

Further analysis was performed on a region of TM similar to that shown in Figure 1B but at a higher optical zoom. A region of TM was flat-mounted and visually sectioned by 2PM at 1 µm intervals to a depth of 75 µm. This depth should encompass the area of JTM and the IWSC. The AF signal of the multiple z-sections were imaged and then computer modeled to show the 3D-aspect of these structures (Figure 3). Snapshot images of the 3D projection shown in Figure 3 are taken at 45° intervals, as rotated about the y-axis. The aqueous humor face of TM (Figure 3; 0°) shows numerous collagen fibers by AF as well as an amorphous collagen structure (black arrow). Rotation of the 3D structure by 180° shows an open pore-like structure (white arrow) that bridges the area of JTM and IWSC. The non-fibrous structure present at the aqueous surface can be viewed through it. This structure is similar to pores found within the IWSC by several different electron microscopy studies (reviewed in [11]) that cannot be attributed to artifacts formed during the fixation and processing of the sample [12]. The 3D animation of the multi-layer reconstruction is present in Figure 4.

**Figure 5:**
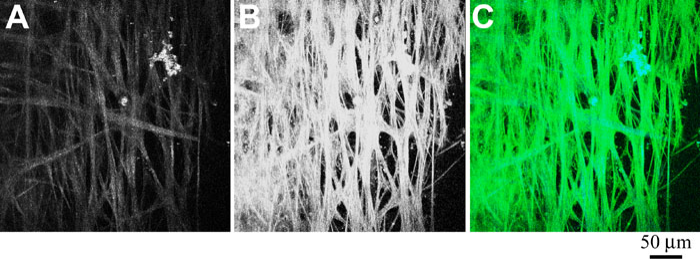
Second-harmonic generation (SHG) and AF of TM region of a human eye from a 73-year-old donor. A section of a human eye was flat-mounted with the anterior chamber facing the microscope objective. Images represent a projection of the multiple z-sections flattened into a single plane. **A**: The SHG emission (388 nm to 409 nm) collected from an 800 nm excitation of TM. **B**: AF collected simultaneously as described in Figure 1. **C**: Merged image of SHG (blue) and AF (green) emission. Black scale bar=50 µm.

**Figure 6:**
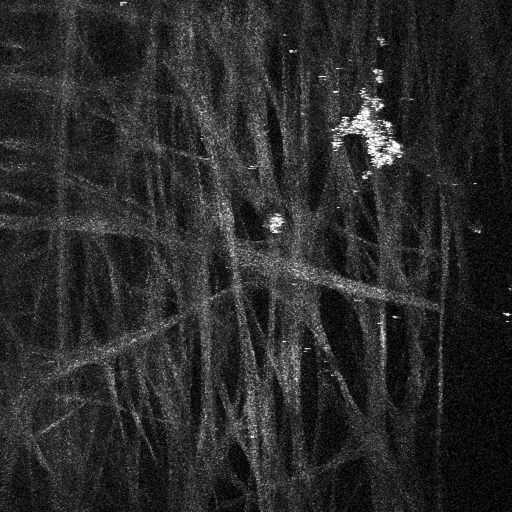
A 3D reconstruction of the TM region from Figure 5. The region of TM shown in Figure 5 was imaged at 0.5 µm intervals (for a total of 100 z-sections). For this animation, only the SHG signal was projected into a 3D-animation. The animation begins with a view of the aqueous face of the TM, with rotation about the y-axis. While the SHG signal is weaker than the AF signal (animation in Figure 4), it shows qualitatively the same interwoven 'beam' structures surrounded by fluid-filled spaces.

We also performed simultaneous imaging of AF and second harmonic generation (SGH) in a third region of human TM. SHG and AF emission windows were collected using the META spectral detector as described in the methods. The TM was flat-mounted and visually sectioned by 2PM at 0.5 µm intervals to a depth of 50 µm and then computer modeled into a single-plane projection (Figure 5). Figure 5A and Figure 5B show the SHG and AF fluorescence, respectively. Although the SGH signal is comparatively weaker than the AF, these two signals are qualitatively the same when overlapped in Figure 5C (blue=SHG, green=AF). Since collagen is the most common non-centrosymmetric macromolecule in the TM, the SGH signal is highly suggestive that the structures seen by AF (Figure 1 and Figure 3) are in fact collagen fibers. In these images of the TM, the majority of collagen fibers of the TM appear as smooth bundles of between 10 and 20 µm, although the occasional ~1 µm collagen fibers is visible. These bundles have a fairly consistent diameter over short distances, but over longer distances (>250 µm) commonly split or join other bundles. The end result is a meshwork of collagen interwoven with varying-sized regions of non-fluorescent signal, which we assume to be fluid spaces. The 3D animation of the SHG projection shown in Figure 5A is presented in Figure 6.

**Figure 7:**
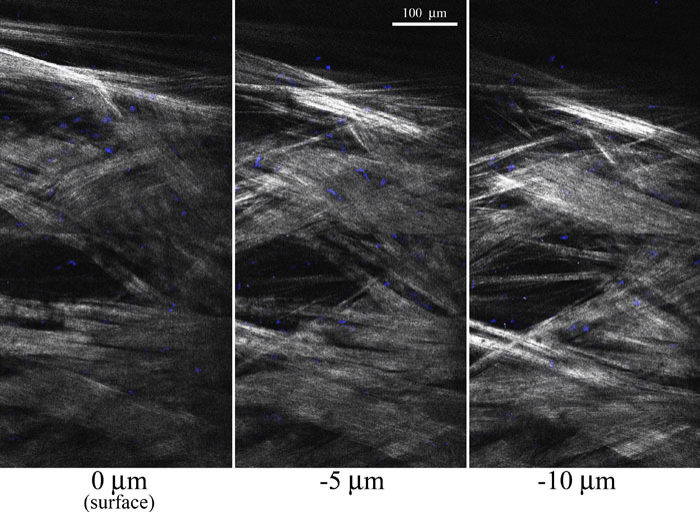
2-photon excitation fluorescence (2PEF) and second harmonic generation (SHG) of TM region of a human eye from an 88-year-old donor. A section of a human eye was labeled with the cell-permeable nuclear stain (Hoechst 33342) then flat-mounted with the anterior chamber facing the microscope objective. A 2PEF emission window for Hoechst (473 nm to 505 nm) and a SHG emission window (388 nm to 409 nm) was collected from an 800 nm excitation. Multiple tiled scan were performed at the aqueous humor surface of the TM (0 µm) and at −5 and −10 microns below the surface. White=SHG signal, blue=Hoechst. White scale bar=100 µm.

**Figure 8:**
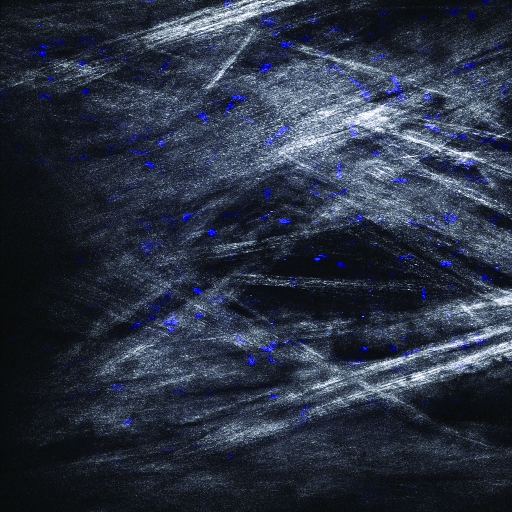
A 3D reconstruction of a TM region from Figure 7. The area of TM shown in Figure 7 that encompasses the large ‘fluid space’ near the center was imaged at 0.5 µm intervals. A total of 85 z-sections were projected into a 3D-animation, with the 2PEF from the Hoechst-labeled nuclei shown in blue and the SHG from collagen in white. The animation begins with a view of the aqueous face of the TM, with rotation about the y-axis. The pattern of blue-stained nuclei indicates that TM endothelial cells are evenly distributed throughout the collagen bundles of this region.

Toward the goal of imaging TM cells within unfixed tissue, we measured endothelial cells in the TM region using the fluorescent nuclear stain (Hoechst 33342). The dye was injected into the anterior chamber of an intact donor eye. The eye was then opened, and a section of TM was flat-mounted with the aqueous-surface facing the microscope objective. We imaged the TM cell nuclei (Figure 7; shown in blue) by 2PEF of the fluorescent dye. The collagen fibers within the TM were imaged simultaneously by SHG (Figure 7; white). This figure represents a z-planes created by stitching together several overlapping images. At the magnification used in this measurement, the long collagen fibers are just visible, organized into multiple bundles of parallel strands. There are regions of both tightly overlapping collagen bundles (Figure 7; imaged by SHG, shown in white). There are also fluid-filled regions (up to ~100 µm in diameter) extending from the surface of the TM (Figure 7; 0 µm) toward the JTM (−5 µm and −10 µm). These open regions are not to be confused with the SC which would be much larger in size and oriented perpendicular to the imaging plane. Finally, the Hoechst staining (blue) shows the sparse distribution of TM endothelial cells adhering to the collagen bundles throughout the TM region. A 3D animation of the SHG signal from a 40 µm deep region of the central fluid-space of Figure 4 is presented in Figure 8.

## Discussion

2PM includes both two-photon excitation fluorescence (2PEF) and second harmonic generation (SHG). 2PEF is very similar to traditional fluorescence, except two photons of a lower energy are simultaneously absorbed to excite a fluorophore. The excited fluorophore subsequently fluoresces a single photon of the appropriate emission wavelength. 2PEF occurs with both endogenous and exogenous fluorophores. 2PEF can be used to excite endogenous biologic chromophores such as NAD(P)H, collagen, elastin, and melanin [13-15]. Imaging by this method is often referred to as two-photon excitation autofluorescence (2PAF) or autofluorescence (AF) in short, since the fluorescence results from the intrinsic properties of these molecules and not from any external fluorescent label. Another nonlinear process that occurs with 2PM is second harmonic generation (SHG). SHG can only occur with non-centrosymmetric (asymmetric) macromolecular structures. Collagen fibers can simultaneously “scatter” two lower-energy photons as a single photon of twice the energy. SHG signal occurs at a distinct wavelength (half the excitation wavelength) and can be separated from tissue autofluorescence using a spectral detector.

1PM uses excitation wavelengths in the visible range (400–600 nm), which undergo significant optical scattering and material absorption in biologic samples. This limits 1PM visualization to within 100 µm of the surface of the tissue (reviewed in [16]). 2PM is therefore much better suited to deep tissue imaging. For example, living skin has been imaged by 2PM to a depth of 350 µm by visualizing the AF of the skin’s extracellular matrix and melanin [17]. The resolution in this study was determined to be 0.5–1 µm lateral by 3–5 µm axial, which is on par with typical a 5 µm thick histological section. Because of its great penetrating ability, 2PM has been used to successfully image the intact human cornea [18] as well as flat-mounts of human retina and retinal pigment epithelium (RPE) [19,20]. Furthermore, 2PM has been used to image the RPE and retina within the intact eye of rodents [21]. A recent publication performed the first simultaneous 2PEF and third harmonic imaging on the cornea to detect elastin and collagen structures, and also showed the collagen and elastin structures of the TM [22]. The authors were able to discern the prominent Schwalbe’s line, but they did not perform the deep-tissue imaging that is presented here, and also did not perform parallel imaging with a nuclear stain to simultaneously detect TM endothelial cells.

In this study, 2PM was used to image the native TM region of the human eye by AF and SHG. This method has the advantage over light microscopy of histological sections or EM on ultra-thin sections by being performed on unprocessed tissue. This eliminates distortions within the tissue due to infusion of fixatives, shrinkage of tissue due to alcohols, and changes to fine tissue morphology that can occur with heat-infusion of paraffin. There is presently no evidence that image artifacts are created from the hardware or software that we discuss in this report. In fact, since the software is calibrated to the optical properties of the objective lens, the software should correct for any distortions introduced by the imaging hardware. The TM was imaged en face by SHG and the ‘meshwork’ was found to have fluid spaces of non-uniform size, in confirmation of the organizational structures shown by quick-freeze deep etching EM of Gong et al. [10]. Additionally, we were able to detect structures consistent with pores found in the IWSC (reviewed in [11]). In contrast 2PM allows additional capabilities in comparison to EM by allowing fluorescent staining to highlight certain cell structures or proteins, and by allowing chemical-specific imaging by auto-fluorescence. The ability of z-resolution imaging to obtain full 3D structures of unprocessed samples is another key advantage.

The imaging presented here represents microscopy starting from the aqueous humor face of the TM toward the JTM region, with the microscope objective located within a millimeter of the surface of the tissue. We realize that this would not be amenable to the clinical imaging of patient TM, but we believe these 2PM images validate this method and represent a great leap forward in understanding the native structures within the TM. These findings have served as the foundation for performing current studies investigating 2PM imaging techniques in perfused whole human eye specimens using proprietary systems.

The hallmark indicator of glaucoma, elevated intraocular pressure (IOP), is believed to result from dysfunction in the anterior fluid drainage system of the eye. It is clear that surrogate metrics for glaucoma, such as IOP, are not fool-proof in diagnosing disease. Patients with elevated IOP often do not develop glaucoma and patients with glaucoma do not necessarily have high IOP [23,24]. Other metrics are needed for the care of glaucoma patients to identify cellular dysfunction in vivo rather than using the surrogate metrics that have limited diagnostic sensitivity and specificity. This study shows that 2PM is useful for imaging tissues responsible for the regulation, or dysregulation, of aqueous humor outflow from the eye. Further studies are needed to explore the safety of implementing this imaging device in clinical practice, and to move forward with the creation of novel methods to bypass the limbal tissues that shield the drainage system from the reach of currently available 2PM imaging microscopes.

## Supplementary Material

Supporting Movie

## Supplementary Material

Supporting Movie

## Supplementary Material

Supporting Movie

## Supplementary Material

Supporting Movie
